# Non-Retroviral Fossils in Vertebrate Genomes

**DOI:** 10.3390/v3101836

**Published:** 2011-10-10

**Authors:** Masayuki Horie, Keizo Tomonaga

**Affiliations:** Laboratory of Human Tumor Viruses, Department of Viral Oncology, Institute for Virus Research, Kyoto University, 53 Kawahara-cho, Shogoin, Sakyo-ku, Kyoto 606-8507, Japan; E-Mail: tomonaga@virus.kyoto-u.ac.jp

**Keywords:** viral fossil, endogenous non-retroviral virus-like elements, non-retroviral endogenization, non-retroviral integration, exaptation

## Abstract

Although no physical fossils of viruses have been found, retroviruses are known to leave their molecular fossils in the genomes of their hosts, the so-called endogenous retroviral elements. These have provided us with important information about retroviruses in the past and their co-evolution with their hosts. On the other hand, because non-retroviral viruses were considered not to leave such fossils, even the existence of prehistoric non-retroviral viruses has been enigmatic. Recently, we discovered that elements derived from ancient bornaviruses, non-segmented, negative strand RNA viruses, are found in the genomes of several mammalian species, including humans. In addition, at approximately the same time, several endogenous elements of RNA viruses, DNA viruses and reverse-transcribing DNA viruses have been independently reported, which revealed that non-retroviral viruses have played significant roles in the evolution of their hosts and provided novel insights into virology and cell biology. Here we review non-retroviral virus-like elements in vertebrate genomes, non-retroviral integration and the knowledge obtained from these endogenous non-retroviral virus-like elements.

## 1. Molecular Fossils of Ancient Viruses

We can learn about ancient organisms from their fossil records. They are the evidence of the existence of organisms in the past and have given us important and interesting knowledge regarding ancient living things; for example, their features and evolution. Thus far, fossils of the bodies of many organisms have been found but those of viruses have not been discovered. However, a particular type of virus, reverse transcribing RNA viruses (*i.e.*, retroviruses), are known to leave molecular fossils in the genomes of their host species [1]. Retroviral genomes integrate into the host chromosome during their replication using the virus-encoded reverse transcriptase and integrase. If a viral genome becomes integrated into the chromosome of host germ cells, the viral sequence will become part of the genome of the offspring of the host and the viral sequences will then be inherited vertically in a Mendelian fashion. This process is called “endogenization” and endogenized retroviruses are called endogenous retroviruses (ERVs). ERVs comprise approximately 8% and 10% of the human and mouse genomes, respectively [2,3]. Interestingly, several proteins encoded by ERVs have been reported to play important roles in host physiology, for example, placentation and anti-retroviral function [4–8]. Thus, ERVs are not only the evidence of past retroviral infections but provide information regarding the co-evolution of retroviruses and their hosts [1]. On the other hand, until recently, germ line integration of non-retroviral viruses, namely RNA, DNA and reverse transcribing DNA (RT-DNA) viruses, has not been found in vertebrate genomes.

## 2. Discovery of Non-Retroviral Virus-like Elements

### 2.1. Endogenous RNA Virus-like Elements

Although several RNA viruses had been reported to integrate into their hosts’ genomes [9–11], no germ line integration had been demonstrated in vertebrate genomes until we and another group independently discovered elements derived from the nucleoprotein (N) gene of an ancient bornavirus, which were designated as endogenous Borna-like N (EBLN) elements [12–14] (Table 1). Bornaviruses are non-segmented, negative strand RNA viruses belonging to the order *Mononegavirales*. Bornaviruses are unique RNA viruses in that they readily establish non-cytopathic, persistent infections in the nuclei of host cells [15]. In our analyses, EBLNs were detected in several species of mammals including humans. In addition, bornavirus transcription regulatory signal-like sequences are located on either side of EBLNs. Some EBLNs seem to be orthologous between species, which enables us to estimate their minimum age. Because presence of orthologous viral elements at the same loci in two or more different species shows that integration occurred in their common ancestor, integration age can be deduced from the estimated divergence time of animals. Phylogenetic and synteny analyses suggested that EBLNs are orthologous among anthropoidea, therefore these EBLNs were estimated to have been generated by integration of an ancient bornavirus N gene at least 40 MYA. Thus, Bornaviruses are the first viruses known to coexist with animals in the prehistoric age [12]. Interestingly, the thirteen-lined ground squirrel EBLN is located in the cluster of exogenous bornaviruses in the phylogenetic tree, suggesting that formation of squirrel EBLN was a very recent event.

At approximately the same time, non-retroviral RNA virus-like elements were sought systematically by several investigators [13,14], who found additional EBLNs and endogenous elements derived from the bornavirus matrix (M), glycoprotein (G), which is involved in virus entry [16–18], and RNA-dependent RNA polymerase (L) genes in the genomes of vertebrates. Among them, EBLNs are the most widespread endogenous elements in vertebrate species: Primates, rodents, carnivore, bats, afrotheria, insectivore, marsupials and lamprey. Because EBLNs and their flanking sequences are readily aligned in afrotheria species, afrotheria EBLNs also seem to be orthologous. Hence, ancient bornavirus may have coexisted with afrotheria species before their divergence, namely 93 MYA [14].

In addition to bornaviruses, several RNA viruses-related elements, filovirus, nyamaninivirus and tamana bat virus (TBV), have been found in vertebrates [13,14,19] (Table 1). Filovirus-like elements were detected in several mammalian species. Filoviruses, which include Ebola and Marburg viruses, are also members of the order *Mononegavirales*. Endogenous filovirus-like elements related to the major nucleocapsid (NP), polymerase cofactor (VP35), envelope glycoprotein (G) and polymerase (L) genes have been detected in several mammalian genomes. All filovirus NP-related sequences identified consist of fragments showing similarities only to the N-terminal region of the filovirus NP gene [13,19], which is known to be conserved among filoviruses and paramyxoviruses [20,21]. This bias may be attributed to the conservation of this region during viral evolution. Alternatively, these fragments may have conferred some advantageous phenotype to their hosts.

Nyamaninivirus/midwayvirus L and TBV NS3-related sequences were found in Zebrafish and Medaka, respectively. Nyamaninivirus and midwayvirus also belong to the *Mononegavirales* [22]. Because TBV is a member of the flaviviruses, the TBV-like element is the only element derived from a positive stranded RNA virus [23].

Intriguingly, some of endogenous RNA virus-like elements retain large ORFs. Above all, it is surprising that human EBLN-1 and -2 contain significant large ORFs: The EBLN-1 ORF has a coding capacity of 366 amino acids (aa), which is equivalent to bornavirus N (370 aa) in length and the EBLN-2 ORF consists of a 5′ 47 codon fragment derived from cellular sequences and 225 codons homologous to bornavirus N. These elements were shown to be expressed as mRNA in several human cell lines [12], suggesting that they are functional in human cells. Two independent investigators inferred that EBLN-1 has exapted in the primate genome, but EBLN-1 has not retained an intact ORF in all extant primate species, which indicate that selection to maintain the ORF may have been lost recently [13,14]. Our recent analyses revealed that no natural selection was detected after the divergence of old world and new world monkeys, supporting these conclusions [24]. Interestingly, EBLNs tend to be found in animals which are resistant to bornavirus infection or do not develop severe symptoms with bornavirus infection [13]. Excessive amounts of the bornavirus N proteins inhibit its replication, presumably because inappropriate molecular ratio of viral ribonucleoprotein components disturbs viral polymerase activity [25,26]. Therefore, it is speculated that EBLNs have served as restriction factors against ancient bornavirus infection, resulting in the extinction of certain lineages of bornaviruses which infected the ancestors of the animals harboring these EBLNs. Then, the extinction may have led to the relaxation of functional constraints. Interestingly, however, the protein encoded by human EBLN-2 was detected and identified as an interaction partner of several other proteins [27]. These observations indicate that EBLNs may have lost their original functions during evolution and then human EBLN-2 might be acquiring a novel function in our cells.

Some endogenous filovirus-like elements also contain a significant large ORF and are found in the EST database. It was documented that expression of partial N-terminal sequences of Ebolavirus inhibits its replication through dominant-negative effect on wild type NP [20]. Indeed, filovirus NP-related sequences show similarities only to the N-terminal region of filovirus NP, suggesting that filovirus-like elements also have served as anti-virus factors.

### 2.2. Endogenous DNA Virus-like Elements

Endogenous DNA virus-like elements were also searched for systematically, leading to the discovery of parvovirus, dependovirus and circovirus-related endogenous elements in several animal genomes [14,28,29] (Table 2). All three viruses are single-stranded DNA (ssDNA) viruses which replicate in the host cell nucleus. Among the ssDNA virus-related sequences, the most widespread elements are dependovirus-like elements and the second most are parvovirus-like. Both parvovirus and dependovirus belong to the family *Parvoviridae* and their genomes consist of two major ORFs, *rep* genes encoding replicase proteins (Rep/NS) and *cap* genes encoding capsid proteins (Cap/VP), flanked by inverted terminal repeats (ITRs) at either end, which form hairpin structures essential for genome replication. Both *rep*- and *cap*-related sequences were detected as endogenous parvoviral and dependoviral elements. Interestingly, many of the *rep* and *cap*-like sequences are positioned close together, which indicates that these elements are derived from viral genomic DNA, rather than viral RNA transcripts. Among the *Parvoviridae*-like elements, orthology between dependoviral elements in the dog and panda is inferred by synteny analysis, suggesting that the minimum age of *Parvoviridae* is 42 million years [28].

Circoviruses have small circular DNA genomes which also contain two major genes, *rep* and *cap*. Endogenous elements related to circovirus have been detected in five vertebrate species. Curiously, most of the endogenous circovirus-like elements identified are derived from only the *rep* gene, except for that of the opossum, suggesting that circovirus *rep* genes have been more conserved than the *cap* gene during evolution or have conferred some advantage to the host species. Synteny analysis suggested that endogenous circoviral elements in dog and panda are orthologous, which indicates that these elements were generated before their divergence, namely 42 MYA [28]. In fact, endogenous circoviral elements in three species of carnivora (cat, dog and panda) may be orthologous, although synteny analysis with cat genome was not carried out due to the preliminary assembly and short contigs of cat genome. Therefore, circoviral integration may have occurred at least 55–68 MYA [14,28].

Some of the ssDNA virus-like sequences retain significant large ORFs. For example, elephant endogenous dependovirus-like element has an ORF equivalent to an intact *rep* gene. Furthermore, some of these are detected in the EST database. These elements might also serve some functions in their host.

### 2.3. Endogenous RT-DNA Virus-like Elements

Comprehensive analyses of endogenous viral sequences revealed that endogenous sequences which are related to hepadnaviruses, RT-DNA viruses, also exist in animal genomes [14,30] (Table 2). Hepadnaviruses have partially double-stranded circular DNA genomes and replicate in the cell nucleus. Two independent groups detected endogenous duck hepatitis B virus (DHBV)-related sequences in the genome of the zebra finch (*Taeniopygia guttata*), which were designated as “endogenous zebra finch HBVs: eZHBVs”. Fifteen eZHBVs were shown to be interspersed into 10 chromosomes of the zebra finch. Gilbert and Feshotte also detected the elements orthologous to two of the eZHBVs (eZHBVa and eZHBV1) in the genomes of several other bird species by PCR with primers on flanking regions of these insertions, which enable them to calculate the minimum age of eZHBVs [30]. By two different methods, eZHBVa and eZHBV1 were estimated to have endogenized at least 19 MYA. In addition, long-term substitution rates of hepadnaviruses were also calculated (2.15 × 10^8^ to 6.8 × 10^8^ substitutions/site/year), which revealed that the long-term rates are 1000-fold lower than the short-term rates estimated based from the hepadnaviruses circulating currently [30]. Several hypotheses were proposed to explain the time dependency of substitution rates. Variation in the fidelity of reverse transcriptase and/or replication rate could in part explain the difference between short- and long-term evolution. Fidelity of reverse transcriptase of ancient hepadnaviruses might have been higher than that of modern hepadnaviruses. It is also conceivable that the replication rates of hepadnavirus have been low for most of the evolutionary history of hepadnaviruses. Purifying selection may also be involved in the time dependency of substitution rate. Approximately 60% of the HBV genome codes for two or more overlapping ORFs, where there are few synonymous sites. This suggests that purifying selection has strongly affected the long-term substitution rate of hepadnavirus. Lastly, mutational saturation may hinder the accurate estimation of long-term hepadnaviral evolution.

## 3. Non-Retroviral Integration

There are two essential steps for endogenization of exogenous viruses: infection of the host’s germ cells and integration into the host’s nuclear chromosome. Regarding viral tropism, it is no surprise that the ancient viruses which are the origins of endogenous virus-like elements could infect germ cells, even though related extant viruses cannot infect them, because viruses exhibit different host cell tropisms even within the same family. On the other hand, integrations of non-retroviruses have been reported occasionally, which enable us to appreciate the process of endogenization of ancient non-retroviral viruses. In addition, evidence suggesting the mechanisms of past viral integrations may be observed in several endogenous non-retroviral virus-like elements. Here, we review the non-retroviral chromosomal integration reported thus far and discuss the mechanisms of ancient viral integration events.

### 3.1. Integration of Extant RNA Viruses

Because non-retroviral RNA viruses do not encode a reverse transcriptase or integrase, these RNA viruses are generally believed not to integrate into the genomes of their hosts under physiological conditions. Nonetheless, several RNA viruses have been reported to be reverse transcribed and/or integrated into their hosts’ genomes. Most of the reports described only the phenomenon of viral reverse transcription and/or integration, whereas the integration mechanisms of Lymphocytic choriomeningitis virus (LCMV) and Borna disease virus (BDV) were inferred from biological experiments.

LCMV is a segmented, ambisense RNA virus which belongs to the family *Arenaviridae*. Geuking *et al.* demonstrated that partial genomic sequences of LCMV were integrated into host chromosomes *in vitro* in infected cell culture and *in vivo* in infected mice by recombination with the murine intracisternal A-particle (IAP) element, an LTR-retrotransposon [11]. Transfection of plasmids encoding a functional IAP led to the formation of LCMV cDNA in cells in which LCMV cDNA could not be detected under normal conditions. Interestingly, plasmids encoding HIV-1 *gag-pol* also supported the formation of LCMV cDNA, albeit much less efficiently than IAP, whereas LCMV cDNA was not detected in infected CCL158 cells, a guinea pig cell line which shows a high level of RT activity [10]. These findings suggest that combination of virus-host (retroelements) is a critical factor for LTR-retrotransposon-mediated integration of RNA viruses. Although an immunological function was speculated [10], the physiological significance of IAP-mediated LCMV reverse transcription and integration remains unclear. No endogenous element of LCMV has been found in the genomes of living things.

In the case of BDV, it was shown that the mRNA of BDV is reverse transcribed and integrated into the host genome [12]. BDV cDNA was not detected with primers spanning the boundaries of BDV transcription units. In addition, sites of integration in the host chromosome were determined by inverse PCR, which revealed that many of the clones identified have poly-A tails and target site duplications (TSDs). These observations suggested strongly that long interspersed nuclear element-1 (LINE-1), a non-LTR retrotransposon, is involved in the reverse transcription and integration of BDV mRNA. Indeed, LINE-1 occasionally participates in the formation of processed pseudogenes of cellular mRNA *in trans* [31,32]. Thus it is no wonder that the reverse transcriptase of LINE-1 catalyzes the reverse transcription of BDV mRNA. It remains unclear whether or not LINE-1 recognizes BDV mRNA preferentially and further studies are needed to elucidate the mechanisms of LINE-1-mediated BDV integration.

Taken together, reverse transcription and integration of LCMV and BDV are achieved with the help of enzymes encoded by cellular retroelements. LCMV and BDV have common biological features in that both viruses establish persistent infections. Although it remains unclear whether this feature is critical for integration of RNA viruses, persistent infection must make it easy to detect cDNA molecules of RNA viruses in infected cells, in contrast to acute infection. Because LINE-1 recognizes mRNA nonspecifically and forms a ribonucleoprotein complex in the cytoplasm, all viruses, independent of their replication sites, theoretically have the potential to be integrated into the chromosomal genome by LINE-1-mediated mechanisms. Furthermore, there may be virus-host (host’s retrotransposon) combination in which viral integration occurs, as in the case of LCMV and the mouse (IAP element). Thus, it is likely that other RNA viruses also may integrate via retroelement-mediated mechanisms.

Importantly, these observations raise the possibility that non-retroviral RNA viruses act as mutagens [11,33,34]. The risk of somatic integration of RNA virus vectors should be evaluated, as for LCMV and BDV.

### 3.2. Integration of Ancient RNA Viruses

According to BDV integration, human EBLNs have typical structures of LINE-1-mediated integrations, namely TSDs and 3′ poly-A sequences [12,13]. Interestingly, LINE-1 has been reported to have been highly active in ancient primates 40–50 MYA [35], which is in agreement with the time when anthropoid EBLNs are estimated to have been generated. These observations strongly suggest that EBLN may be generated by LINE-1-mediated integration. Some endogenous filovirus-like elements also have TSDs and 3′ poly-A sequences, indicating that LINE-1 may also have been involved in their generation [13]. Although the mechanisms for the generation of other non-retroviral RNA virus-like elements remain unknown, their integrations may have been mediated by retrotransposition or, alternatively, by co-infection with retroviruses. Intriguingly, most of the endogenous non-retroviral RNA virus elements identified thus far are derived from negative strand RNA viruses, which suggests that ancient negative strand RNA viruses had some predisposition for endogenization.

### 3.3. Integration of Extant DNA Viruses

Some DNA viruses have been reported to integrate into their host’s genome despite the absence of a virus-encoded integrase.

Chromosomal integration of adeno-associated virus 2 (AAV2), a dependovirus, has been well studied. AAV2 preferentially integrates into a specific locus in human chromosome 19q.13.4, which contains a binding site for the AAV2 Rep protein and a Rep-specific nicking site. Rep proteins recognize this site and mediate a synapse between viral and host sequences, leading to site-specific recombination [36]. In addition to Rep-dependent site-specific integration, an AAV vector was also reported to integrate into chromosomal DNA at random, presumably by Rep-independent non-homologous end joining (NHEJ) [37]. Surprisingly, AAV integrants were detected in human testicular tissue [38]. Thus, current AAV has the potential to endogenize into host species. It was also reported that minute virus of mice, a member of the genus parvovirus, integrates into the host chromosomes, presumably via mechanisms similar to Rep-independent integration of AAV [39].

Several herpesviruses have been reported to integrate into the host genome. Among them, integration of human herpesvirus 6 (HHV-6) has been well studied. HHV-6 specifically integrates into telomere regions of human chromosomes, presumably via homologous recombination between human telomeres and the viral direct repeats [40]. Intriguingly, several reports showed that integrated HHV-6 can be transmitted vertically via germ cells [41,42].

### 3.4. Integration of Ancient DNA Viruses

In many of the endogenous parvoviral and dependoviral elements, *rep* and *cap*-related sequences are found in close proximity, indicating that they originate from genomic DNA rather than RNA transcripts [28]. No ITR was found except for degraded ITR-like sequence observed in microbat dependoviral elements, which could be explained by sequence degradation or the absence of ITRs in ancient parvoviruses [28]. Regarding circovirus-like elements, most consist of only *rep*-related sequences. In the opossum genome, however, a short, *cap*-like sequence is adjacent to *rep* in the opposite orientation, which is similar to the organization of circovirus genome. This suggests that it is derived from viral genomic DNA [28]. In the replication cycles of these ssDNA viruses, dsDNA molecules are generated as intermediate products in the host cell nucleus. These dsDNA molecules may have been inserted by host recombination mechanisms such as NHEJ.

### 3.5. Integration of Extant RT-DNA Viruses

RT-DNA viruses have unique replication cycles. While they have DNA as their genome in the virions, the replication system is quite different from that of DNA viruses in that RT-DNA viruses replicate via RNA intermediates. Specifically, after infecting host cells, viral genomic DNA is transcribed into RNA, and then the RNA is reverse transcribed into DNA. Hepadnaviruses, RT-DNA viruses, are well known to integrate into the host’s genome, although integration is not a step in the replication cycle. While the precise mechanism of hepadnaviral integration remains unclear, it has been reported that viral linear double-stranded DNA can be integrated into the chromosomes during the repair of genomic DNA double-strand breaks via NHEJ [43]. Surprisingly, hepadnaviral integration was observed not only in hepatocytes but also in spermatozoa [44], which suggests that extant HBV also has the potential to make generate endogenous elements.

### 3.6. Integration of Ancient RT-DNA Viruses

In the case of eZHBVs, none have a TSD and poly-A tail [30]. Furthermore, as described above, it was demonstrated experimentally that hepadnaviral integration can occur during the repair of host genomic DNA double-strand breaks via NHEJ. These observations suggest that eZHBVs may have been generated by NHEJ or other recombination mechanisms, rather than retrotransposon-mediated integration.

## 4. Perspective

The study of endogenous non-retroviral virus-like elements has just begun. Nonetheless, many germ line integrations of non-retroviruses have been discovered in vertebrate genomes in a period of less than two years, which not only confirms the coexistence of non-retroviral viruses and vertebrate animals in the prehistoric age but also provides novel insights into virology and cell biology, such as integration of BDV and long term evolution rates of hepadnaviruses [12,30]. In addition, it was demonstrated that non-retroviruses have contributed to the evolution of vertebrates. These elements are also found in invertebrate genomes [14,45,46].

It remains unclear why these elements reached fixation. They may have been fixed incidentally or they may have been exapted in their host genomes, which conferred some survival advantages to their hosts. Although many observations suggest that several endogenous non-retroviral viral elements have been exapted, none has been demonstrated to have a function. Because natural selection should have operated on a gene encoding a functional protein, evolutional analyses such as *d*_N_/*d*_S_ examination should aid our understanding. In addition, biological examination should be conducted to understand the significance of current endogenous viral elements in their hosts. It is possible that these elements have some function, not only as proteins but also as non-coding RNA, targets of miRNA, or regulatory elements of gene expression.

The enrichment of genomic sequence databases will enable us to gain novel information about endogenous viral elements. PCR-based approaches are also useful, as several investigators have shown [16,30]. These studies will give us novel insights into virology, evolutional biology and cell biology.

## Figures and Tables

**Table 1.** Endogenous non-retroviral RNA virus-like elements in vertebrates [13]. “+” and “+/−” show endogenous viral elements with BLAST E-value below 10^−10^ and as high as 10^−5^, respectively. (+) ssRNA: Positive-strand RNA virus, (−) ssRNA: Negative-strand RNA virus, TBV: Tanama bat virus, NV: Nyamaninivirus/Midwayvirus.

	(+) ssRNA	(−) ssRNA								
	TBV	Bornavirus				Filovirus				NV
	*NS3*	*N*	*M*	*G*	*L*	*NP*	*VP35*	*G*	*L*	*L*
Anthropoid		+		+/−						
Tarsier		+					+	+		
Lemur		+	+							
Bush baby		+								
Mouse		+			+					
Rat		+			+					
Kangaroo rat		+/−						+/−		
Squirrel		+								
Guinea pig		+				+/−				
Cow		+/−								
Horse								+/−		
Microbat		+			+	+	+			
Shrew		+				+/−		+/−		
Elephant		+								
Hyrax		+								
Tenrec		+/−								
Opossum		+				+			+	
Wallaby		+/−			+	+	+			
Tetraodon								+/−		
Takifugu					+					
Fugu								+/−		
Medaka	+/−		+/−		+					
Zebrafish								+/−		+
Stickleback								+/−		
Lamprey		+								

**Table 2.** Endogenous DNA and RT-DNA virus-like elements in vertebrates. “+” and “+/−” show endogenous viral elements with BLAST E-values below 10^−10^ and as high as 10^−5^, respectively. ssDNA: Single-stranded DNA virus, RT-DNA: Reverse transcribing DNA virus.

	ssDNA						RT-DNA	
	ParvoV		DependoV		CircoV		HepadnaV	
	*rep*	*cap*	*rep*	*cap*	*rep*	*cap*	*C*	*P*
Baboon			+	+				
Tarsier			+					
Mouse			+	+				
Rat	+	+	+	+/−				
Kangaroo rat			+					
Squirrel				+/−				
Guinea pig	+	+	+					
Rabbit			+	+				
Pika			+					
Alpaca				+				
Cow			+					
Pig			+					
Dolphin			+	+				
Horse			+					
Cat			+/−		+/−			
Dog			+/−	+	+			
Panda			+/−		+			
Microbat			+	+				
Megabat			+					
Armadillo				+				
Elephant			+					
Hyrax		+	+	+				
Tenrec	+							
Opossum	+	+			+	+		
Wallaby	+	+	+	+				
Platypus			+	+/−				
Zebra finch				+			+	+
Frog					+			
Tetraodon			+	+				
Fugu	+	+						
